# Anti‐PD1 or anti‐PD‐L1 antibodies alone or in combination with chemotherapy first‐line treatment of advanced non‐small cell lung cancer

**DOI:** 10.1111/1759-7714.14358

**Published:** 2022-02-24

**Authors:** Xuexia Zhang, Xiaoping Cai, Hao Zheng

**Affiliations:** ^1^ Department Internal Medicine Sixth affiliated Hospital of Wenzhou Medical University, Lishui People's Hospital Lishui PR China; ^2^ Department of Respiratory and Intensive Care Unit Sixth affiliated Hospital of Wenzhou Medical University, Lishui People's Hospital Lishui PR China

To the editor,

Non‐small cell lung cancer (NSCLC) is a major pathological type of lung carcinoma and most cases are at an advanced metastatic stage when first diagnosed. With regard to advanced stage NSCLC, chemoradiotherapy, targeted treatment, and antiangiogenesis therapy are the major treatment scenarios with improved prognosis, especially in targeted treatment for those individuals with driving gene mutations.^1^ However, for driving gene mutation negative or unknown cases, the efficacy of aforementioned target treatment is limited. In recent years, with the development of tumor immunotherapy research, immune‐checkpoint inhibitors (ICIs) mainly referring to programmed death receptor‐1 (PD‐1) and programmed cell death 1 ligand 1 (PD‐L1) have achieved breakthrough progress in the treatment of patients with advanced NSCLC.^2^

Several high quality phase II/III prospective multicenter randomized clinical trials (RCTs) have evaluated the efficacy and safety of ICIs monotherapy or ICIs plus chemotherapy versus chemotherapy for advanced NSCLC.^3^, ^4^ Recently, Brito and colleagues^5^ performed a meta‐analysis by combining the results of open published data relevant to the efficacy and safety of ICI monotherapy or ICIs plus chemotherapy versus chemotherapy for advanced NSCLC. In this meta‐analysis, the authors reported 13 prospective clinical trials which included 7673 NSCLC cases. According to Brito et al., the pooled results demonstrated that anti‐PD1 plus chemotherapy had better outcomes with regard to overall survival (OS) and progression‐free survival (PFS) compared to anti‐PD‐L1 plus chemotherapy. However, the difference was not statistically significant for OS and PFS between anti‐PD1 and anti‐PD‐L1 monotherapy. With regard to the objective response rate (ORR) and treatment toxicity, the difference was also not statistically different. The authors suggested that PD‐1 antibody combined with chemotherapy was superior to anti‐PD‐L1 plus chemotherapy for NSCLC; nevertheless, as monotherapy, both strategies appear to be similar.

Although the results are based on 13 prospective clinical trials, the conclusions need further discussion due to methodical issues and limitations. First, the methodical quality of the included 13 prospective trials was not evaluated. The convincing conclusion is based on the combination of high‐quality research. Without methodological quality evaluation, the combined conclusion needs further discussion. Second, the publication bias of the meta‐analysis was not evaluated although the authors mentioned the publication bias evaluation methods of Begg's funnel plot. Third, the clinical heterogeneity such as histology type, chemotherapy regimen, PDL1 expression, across the 13 included trials was obvious. The significant clinical heterogeneity reduced the reliability of the conclusions and requires subgroup analysis if possible.

In our view, despite the aforementioned limitations, Brito et al. are to be commended for their interesting meta‐analysis with the purpose of evaluating an urgently‐needed and hot topic in advanced NSCLC management. However, we believe that the conclusion of the meta‐analysis will be more convincing and clinically practical if the above limitations are acknowledged.
